# Comparing the prognostic impact of^ 131^I and/or artificial liver support system on liver function failure combined with hyperthyroidism

**DOI:** 10.1530/EC-24-0330

**Published:** 2024-10-07

**Authors:** Danzhou Fang, Shiying Li, Changgu Zhou, Yirui Wang, Gengbiao Yuan, HuiHui Zhang, Maohua Rao

**Affiliations:** 1 Department of Nuclear Medicine, The Second Affiliated Hospital of Chongqing Medical University, Chongqing, China; 2 Department of Infectious Diseases, The Second Affiliated Hospital of Chongqing Medical University, Chongqing, China; 3 Department of CT, People's Hospital of Linshui County, Guangan, China

**Keywords:** ^131^I, artificial liver support system, liver function failure, hyperthyroidism

## Abstract

**Objective:**

Hyperthyroidism, a prevalent endocrine disorder, can lead to complications such as liver failure due to the liver's essential role in thyroid hormone metabolism. The study aimed to elucidate the respective contributions of ^131^I and/or ALSS in managing hyperthyroidism alongside liver failure.

**Methods:**

A retrospective analysis was carried out on 74 patients diagnosed with severe liver failure in the context of Graves' disease. Patients were categorized into three groups: group A (*n* = 34) received ^131^I treatment, group B (*n* = 17) underwent ^131^I and ALSS treatment, and group C (*n* = 24) received artificial liver support system (ALSS) treatment alone.

**Results:**

Throughout the treatment period, the liver function indexes in all groups exhibited a declining trend. The thyroid function of group A and group B treated with ^131^I was significantly improved compared to that before treatment. There was no significant change in thyroid function in group C. After the correction of hyperthyroidism, significant improvements were observed in the liver function of individuals in groups A and B, particularly with more noticeable amelioration compared to group C. After two months of treatment, the efficacy rates for the three groups were 79.41%, 82.35%, and 60.87% respectively. Mortality rates of the three groups were 5.88%, 17.65%, and 36% (*P* < 0.01). Group B, receiving both ^131^I and ALSS treatments, exhibited a lower mortality rate than group C.

**Conclusion:**

In cases of severe liver failure accompanied by hyperthyroidism, prompt administration of ^131^I is recommended to alleviate the adverse effects of hyperthyroidism on liver function and facilitate a conducive environment for the recovery of liver functionality.

## Introduction

Hyperthyroidism, an endocrine disorder affecting roughly 0.2–1.3% of the global population, presents a significant health challenge (1). Graves' disease (GD) is the primary cause of hyperthyroidism. Originating in the 19th century, it was initially recognized as a syndrome featuring thyroid enlargement, heightened activity, accelerated heart rate, and eye-related anomalies (2). The thyroid hormones undergo glucuronidation and sulfation in the liver before being excreted into the bile. They also facilitate bilirubin metabolism by controlling glucuronyl transferase and ligandin, a hepatic transport protein (3). Consequently, hyperthyroidism can result in complications ranging from liver function abnormalities to liver failure. As a central player in the metabolism and transport of thyroid hormones, the liver produces major thyroid hormone transporters and regulates circulating thyroid hormone levels. Moreover, thyroid hormones govern hepatocyte metabolism and bilirubin production via lipid metabolism regulation (4). Hence, any disorder affecting the liver or thyroid can potentially impact the function of the other (5). Management of hyperthyroidism co-occurring with liver failure presents unique challenges. In such instances, personalized and comprehensive treatment strategies become imperative, demanding vigilant monitoring and proactive intervention. The underpinning mechanism of liver failure in hyperthyroid patients involves heightened oxygen consumption due to increased metabolic rates and relative hypoxia around the hepatic lobule vein, which leads to apoptosis and oxidative damage (6). Despite the clinical significance of this interrelation, a consensus on the optimal treatment approach remains elusive. Contemporary hyperthyroidism treatments encompass thyroidectomy, antithyroid drugs, and radioactive iodine (^131^I) therapy (7). However, for those with hyperthyroidism and concomitant liver failure, surgery may not be feasible due to the requirement of near-normal thyroid function, and certain critically ill patients may not be viable candidates for surgical intervention. Antithyroid drugs like propylthiouracil and methimazole serve as the initial pharmacological treatments for Graves' hyperthyroidism (8, 9). Nonetheless, antithyroid drugs can be hepatotoxic, potentially inducing mild to severe liver injury, necessitating their discontinuation in such circumstances. The ^131^I treatment seeks to cause injury and subsequent death of thyroid cells, leading most patients to develop hypothyroidism eventually (10). It is crucial, however, to exercise caution with ^131^I treatment during active Graves' ophthalmopathy, pregnancy, and lactation, given its potential to trigger acute radioactive thyroiditis (ART). ART features the sudden release of stored thyroid hormones into the bloodstream, temporarily exacerbating hyperthyroidism symptoms and potentially sparking a thyroid storm (11). To tackle liver failure, the artificial liver support system (ALSS) has surfaced as a promising treatment approach. ALSS aids in liver function recovery through a series of mechanical, physical, chemical, and biological reactions outside the body (12). Although ALSS treatment has demonstrated promise in removing protein-bound toxins like total bilirubin (TB), direct bilirubin (DB), and total bile acid (TBA) (13), it is not devoid of side effects. The most common adverse reactions include a rapid decrease in blood pressure or heart rate, often coupled with symptoms like chest tightness, nausea, and vomiting. Vasovagal reactions were also reported in a majority of patients (approximately 78%) during their initial ALSS treatment (14).

In spite of the availability of these treatment options, a literature gap persists, especially in comparative studies assessing the efficacy of ^131^I alone, ALSS alone, and ^131^I combined with ALSS for managing GD patients with liver failure. To address this gap, we conducted a retrospective study comparing these three treatment modalities. Our study aims to provide healthcare professionals with evidence-based insights into the relative efficacy of these treatments, thereby aiding them in managing GD patients with liver failure.

## Materials and methods

### Patients and recruitment criteria

We conducted a retrospective analysis on a cohort of 74 GD patients with liver failure selected from 452 individuals who received ^131^I treatment at our hospital from March 2010 to March 2023. The study was approved by the Ethics Committee of the Second Affiliated Hospital of Chongqing Medical University (2023.No112) and was conducted without the need for written informed consent. The diagnosis of GD was primarily based on clinical symptoms, elevated ^131^I uptake, and positive thyroid receptor antibodies (TRAb) (10), while liver failure was identified by prothrombin time activity (PTA) less than 60% of TB concentration over 85.5 mmol/L. Patients with other organ failures, severe infections, a follow-up time of less than 2 months, or coagulation dysfunction from other diseases were excluded from the study. Patients were categorized into three groups: group A (*n* = 34) received ^131^I treatment, group B (*n* = 17) underwent ^131^I and ALSS treatment, and group C (*n* = 24) received ALSS treatment alone.

### Comprehensive medical treatment programs

Propranolol is commonly used as a component of comprehensive medical treatment to manage hyperthyroidism with heart failure. It controls symptoms such as rapid heart rate and palpitations while also reducing cardiac strain. For cases involving hepatitis B with hepatotoxicity, standardized antiviral treatments using drugs like entecavir, tenofovir disoproxil fumarate, or tenofovir alafenamide are critical. These drugs suppress viral replication and mitigate liver damage. Conditions such as primary biliary cholangitis and primary sclerosing cholangitis can be treated with ursodeoxycholic acid, which alleviates clinical symptoms, reduces liver inflammation, and slows disease progression (15). For autoimmune hepatitis, the primary therapeutic approach is immunosuppressive therapy with glucocorticoids to quell the immune response causing liver inflammation. Monitoring of liver function, viral load, and disease progression is key to assessing treatment efficacy and adjusting treatment plans as necessary.

### ^131^I treatment

In groups A and B, patients adhered to a low-iodine diet before receiving ^131^I treatment. We used thyroid ultrasound, ^131^I thyroid imaging, and ^131^I intake measurements to assess thyroid mass, length, and iodine's effective half-life. We then calculated an individualized ^131^I dosage using the following formula: therapeutic radioactivity (MBq) = (thyroid mass per gram of thyroid tissue (g) × ^131^I activity (MBq)) / 24 h ^131^I uptake. The goal of these assessments and calculations is to determine an accurate ^131^I dose for each patient. A low-iodine diet helps reduce iodine levels, thereby increasing the effectiveness of ^131^I treatment by enhancing the thyroid gland's uptake of radioactive iodine. The data from thyroid ultrasound and imaging help us understand the size and structure of the thyroid gland, which assists in determining an individualized dose.

### The model for end-stage liver disease (MELD) score

The MELD score predicts survival rates in patients with liver disease by incorporating the international normalized ratio (INR) of serum bilirubin, serum creatinine, and prothrombin time (PT). The MELD score formula is: 3.78 × ln(serum bilirubin mg/dL) + 11.2 × ln(INR) + 9.57 × ln(serum creatinine (mg/dL)) + 6.43. Clinicians frequently use the MELD score to assess the severity of liver disease and predict short-term mortality. The MELD score’s objectivity and standardization make it a reliable tool for treatment decisions and prioritizing liver transplantation (16).

### ALSS treatment

The treatment involved a systemic infusion of unfractionated heparin to maintain anticoagulant blood status. Patients in groups B and C underwent at least one session of ALSS treatment. Extracorporeal circulation was established by placing a single-needle double-lumen catheter in the patient's right anterior inguinal vein, which allowed for the maintenance of an anticoagulant state through heparin intravenous injection administration. The ALSS device used was the Kawasumi KM-9000 model, with parameters set at a blood flow velocity of 80–130 mL/min, circulating albumin at 80–130 mL/min, and a dialysate flow rate of 500 mL/min. Each session lasted 4–6 h, and the frequency of treatment was adjusted based on the patient's condition, ranging from every 3 to 7 days.

### Follow-up and assessment of efficacy

Regular follow-ups and evaluations of patients' conditions were carried out by consulting hospital records and conducting outpatient follow-ups every 1–2 months after discharge. Liver function evaluation included laboratory indexes such as TB, DB, alanine aminotransferase (ALT), aspartate aminotransferase (AST), and PT. For thyroid function evaluation, the main indexes were free tri-iodothyronine (FT3), free thyroxine (FT4), and thyroid-stimulating hormone (TSH). The therapeutic effect was assessed at the 2-month follow-up based on liver and thyroid function indexes. Treatment outcomes were categorized into three groups: cured, improved, and no response. ‘Cured’ indicated that both liver and thyroid functions returned to normal, or that hypothyroidism was present. ‘Improved’ meant that there was an enhancement in liver function and/or thyroid function. ‘No response’ referred to cases where neither liver nor thyroid functions showed improvement or where death occurred. The effective rate was calculated as the sum of the rates for cured and improved outcomes.

### Statistical analysis

Data analysis was conducted using SPSS 26.0 and GraphPad Prism 8.0. Continuous data were analyzed among the three groups using either one-way analysis of variance or the Kruskal–Wallis test, depending on data distribution. To compare the efficacy of the three treatment methods, paired *t*-tests or Wilcoxon signed-rank tests were used, depending on data distribution. The etiological analysis of the three groups of patients was performed using the chi-square test. The effective rate was calculated as the sum of the rates for cured and improved outcomes. The total effective rate was computed as: ((cured + improved) / total) × 100%. A *P* value less than 0.05 was deemed statistically significant.

## Result

### Patient characteristics

No substantial disparities were observed in demographic data and liver function among the three groups. However, it is notable that the group with the highest TB level was group B, registering approximately 359.79 ± 132.47 μmol/L. The results of the statistical analysis showed significant differences in INR, PT, and PTA among the three groups. Specifically, the values for INR, PT, and PTA indicate that the coagulation parameters in group A were slightly more favorable compared to groups B and C, while groups B and C exhibited similar coagulation profiles (17). Group C exhibited a milder degree of hyperthyroidism compared to the other two groups. The MELD scores of the three groups were 11.59 ± 4.79, 15.03 ± 6.50, and 17.35 ± 5.32, respectively (*P* < 0.01). There were no significant differences in MELD scores observed between groups A and B (*P* = 0.167), as well as between groups B and C (*P* = 0.443). The clinical status of group C was slightly worse than the other two groups (Table 1).

**Table 1:** Baseline characteristics of patients.

	Group A	Group B	Group C	*P*
Sex (male/female)	20/14	9/8	11/13	0.620
Age (year)	44.18 ± 14.47	35.24 ± 14.56	46 ± 14.98	0.057
Major cause of liver failure				
Viral hepatitis	16	9	13	0.99
Drug-induced hepatitis	9	4	6	
Hyperthyroidism-induced	7	3	4	
Other	2	1	1	
FT3 (poml/L)	17.63 ± 11.55	19.31 ± 9.89	8.44 ± 10.75	<0.01
FT4 (poml/L)	71.29 ± 38.28	78.18 ± 21.90	38.08 ± 25.35	<0.01
TSH (Uiμ/ml)	0.01 ± 0.01	0.01 ± 0	0.35 ± 1.2	<0.01
Alb (g/L)	30.90 ± 5.61	31.04 ± 5.49	86.72 ± 161.95	0.985
ALT (U/L)	406.35 ± 469.84	288.18 ± 409.96	433.25 ± 458.13	0.401
AST (U/L)	394.18 ± 522.74	308.82 ± 401.52	407 ± 408.56	0.458
TB (μmol/L)	295.10 ± 170.30	359.79 ± 132.47	294.23 ± 109.84	0.273
DB (μmol/L)	217.57 ± 120.30	253.19 ± 112.16	215.37 ± 76.12	0.462
TBA (μmol/L)	208.56 ± 130.96	281.39 ± 170.16	259.49 ± 129.32	0.270
INR	1.37 ± 0.36	1.91 ± 0.89	1.89 ± 0.75	0.005
PTA (%)	72.03 ± 24.54	54.59 ± 30.51	53.35 ± 31.93	0.003
PT (s)	16.69 ± 3.57	21.65 ± 7.99	21.44 ± 6.49	0.004
MELD	11.59 ± 4.79	15.03 ± 6.50	17.35 ± 5.32	0.001
Number of ALSS treatment		1.94 ± 0.966	2.16 ± 1.519	0.978
^131^I dose (mci)	7.60 ± 2.68	9.64 ± 3.58	–	0.065

Alb, albumin; ALT, alanine transaminase; AST, aspartate transaminase; DB, direct bilirubin; FT3, free tri-iodothyronine; FT4, free thyroxine; INR, international normalized ratio; MELD, model of end-stage liver disease; PTA, prothrombin time activity; PT, prothrombin time; TB, total bilirubin; TBA, total bile acid; TSH, thyroid-stimulating hormone.

### Efficacy of liver function

**Figure 1:**
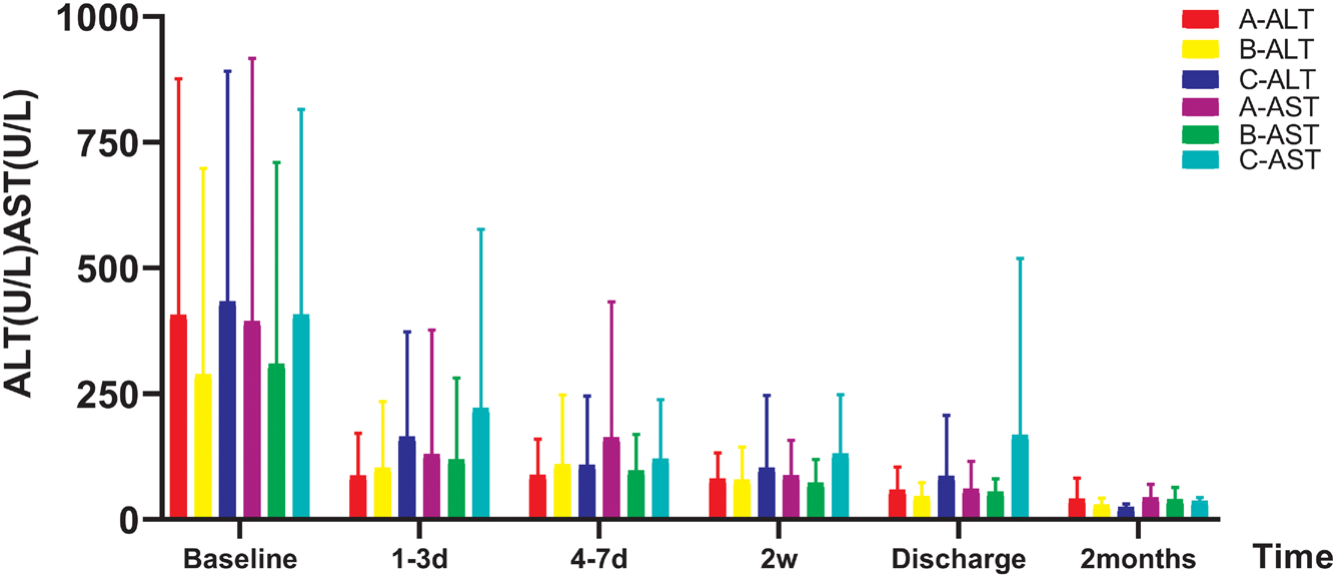
ALT and AST trend chart of three groups of patients before and after treatment. ALT, alanine transaminase; AST, aspartate transaminase; ALT and AST exhibited noticeable improvements at various time points among patients in all three groups, with statistically significant differences observed compared to the baseline levels.

**Figure 2:**
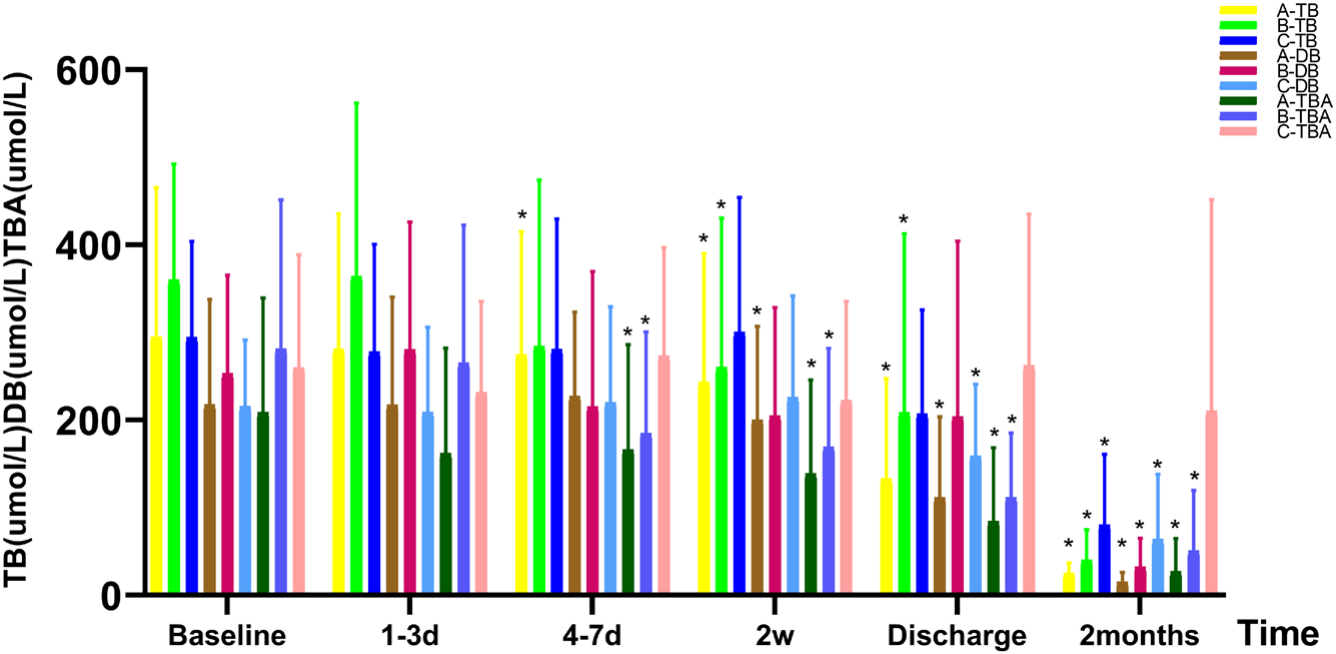
Indicators of TB, DB, and TBA at different time points in three groups of patients. *Represents statistical significance when compared to the baseline. TB, total bilirubin; DB, direct bilirubin; TBA, total bile acid.

ALT and AST exhibited noticeable improvements at various time points among patients in all three groups, with statistically significant differences observed compared to the baseline levels (Fig. 1). Following a span of 2 weeks, there was a substantial reduction in TB levels observed in both group A and group B, with the disparity proving to be statistically significant. Conversely, in group C, a statistically significant discrepancy from the baseline was noted in TB levels at the 2-month follow-up. The most substantial decrease was observed in both group A and group B. In group A, a notable decrease in DB levels commenced at the 2-week mark, while in groups B and C, a significant reduction in DB levels was observed during the follow-up period after discharge. TBA levels in groups A and B exhibited a significant decrease starting at 4–7 days. However, in group C, there was no notable decrease from baseline to discharge (Fig. 2). There were no statistically significant differences in the changes of ALT, AST, TB, and TBA between the three groups after the 2-month treatment compared to baseline. After a 2-month treatment duration, there were statistically significant differences in the DB indices among the three groups when compared to their baseline levels, with group C showing the smallest change (Table 2).

**Table 2:** The effect of three groups on liver function after 2 months of treatment.

Indicators	Group A			Group B			Group C			*P*
	Baseline	Follow-up in 2 months	Difference	Baseline	Follow-up in 2 months	Difference	Baseline	Follow-up in 2 months	Difference	
ALT (U/L)	406.35 ± 469.84	41.08 ± 41.08	424.06 ± 487.68	288.18 ± 409.96	29.06 ± 13.42	302.59 ± 446.61	433.25 ± 458.13	24 ± 7.07	503.29 ± 421.77	0.323
AST (U/L)	394.18 ± 522.74	43.81 ± 26.25	405.71 ± 550.71	308.82 ± 401.52	40 ± 24.16	313.36 ± 438.9	407 ± 408.56	36.57 ± 7.14	453.71 ± 315.7	0.417
TB (μmol/L)	295.1 ± 170.25	25 ± 11.78	258.24 ± 146.17	359.79 ± 132.47	39.97 ± 34.73	288.4 ± 116.78	294.23 ± 109.84	80.46 ± 80.4	156.07 ± 150.66	0.126
DB (μmol/L)	217.57 ± 120.3	15.23 ± 10.78	201.76 ± 108.47	253.19 ± 112.16	32.33 ± 32.66	218.04 ± 82.85	215.37 ± 76.12	63.73 ± 74.46	95.87 ± 90.3	0.028
TBA (μmol/L)	208.56 ± 130.96	27.22 ± 37.67	175.04 ± 123.31	281.39 ± 170.16	50.78 ± 68.81	262.45 ± 172.87	259.49 ± 129.32	210.07 ± 241.86	72.86 ± 201.79	0.38

ALT, alanine transaminase; AST, aspartate transaminase; DB, direct bilirubin; TB, total bilirubin; TBA, total bile acid.

### Efficacy of thyroid function

**Figure 3:**
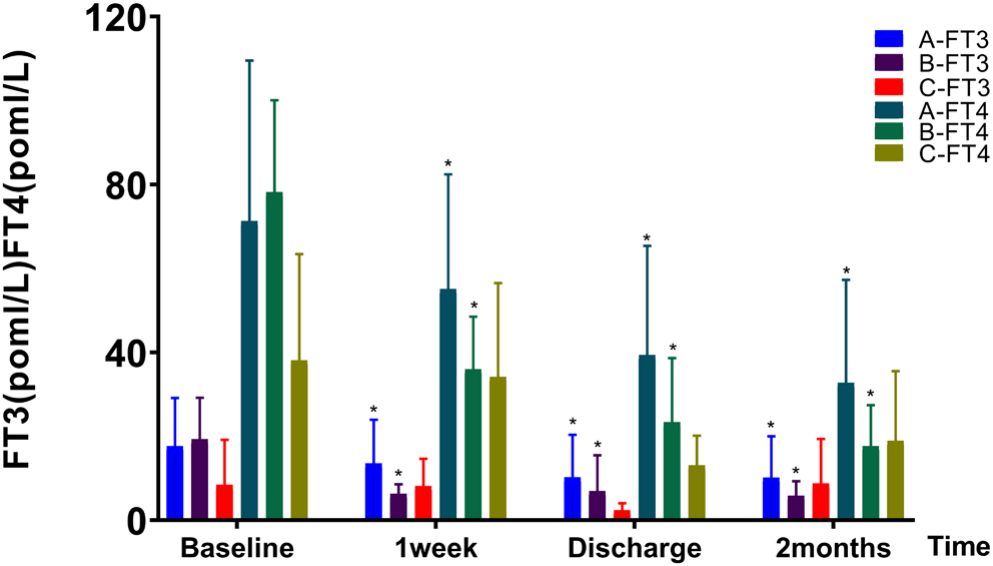
Changes of thyroid function in three groups of patients. *Indicates a statistically significant difference from the baseline. FT3, free tri-iodothyronine; FT4, free thyroxine.

In groups A and B, FT3 and FT4 exhibited a declining trend at various time points post-treatment, revealing significant differences within each group when comparing these levels post-treatment with their respective baseline measurements. In group C, the levels of FT3 and FT4 did not display a significant decrease following treatment; furthermore, these levels were noted to be higher than the baseline measurements during the 2-month follow-up period (Fig. 3).

### Effective rates and mortality

The effective rates of the three groups 1 week after treatment were 64.71%, 70.59%, and 65.22%, respectively. Upon discharge, these rates remained consistent at 70.59%, 70.59%, and 69.57% for the respective groups. However, during the 2-month follow-up, the effective rates of group A (79.41%) and group B (82.35%) were higher than those of group C (60.87%) (Supplementary Table 1, see section on supplementary materials given at the end of this article). The mortality rate in group A was 5.88% (2/34), while in group B, it was 17.65% (3/17). Group C had the highest mortality rate, which was 36%(9/25) (*P* < 0.01) (Supplementary Table 2).

### Costs, daily average cost, and hospitalization time

The number of ALSS treatments received by group B was 1.94 ± 0.234, while group C received 2.24 ± 0.358 treatments (*P* = 0.978). Significant differences were observed in total treatment cost, daily average cost, and hospitalization time among the three groups (*P* < 0.05). Group A, which did not receive ALSS treatment, had the lowest total cost at ¥38203.43 ± 22581.95. On the other hand, group B had the longest average hospitalization time, while group C exhibited the highest average daily cost at ¥3308.03 ± 2063.48 (Supplementary Table 3).

## Discussion

This study presents the first retrospective analysis of three treatment methods for GD and liver failure:^131^I alone, the combination of ^131^I with ALSS, and ALSS alone. The thyroid function of groups A and B, who received ^131^I treatment, demonstrated significant improvement compared to group C, while the liver function of groups A and B did not deteriorate. In the treatment of hyperthyroidism, the selective concentration of ^131^I by functional thyroid tissue leads to subsequent tissue destruction via beta radiation over several weeks. ^131^I therapy is considered safe and well-tolerated for hyperthyroidism (18). Severe hepatotoxicity post-radioactive iodine (RAI) treatment in previously healthy GD patients is rare. Proper patient selection and regular follow-up affirm the safety and effectiveness of RAI treatment, achieving high cure rates (19, 20, 21). Our findings differ from the Chinese guidelines for ^131^I treatment of GD hyperthyroidism in 2021:^131^I treatment results in the release of thyroid hormones stored in the follicles into the blood and increases thyroid hormone levels (22). The treatment strategies of groups A and B, involving ^131^I therapy, effectively target the thyroid gland, mitigating the liver's metabolic burden caused by hyperthyroidism.

In comparison to group C, the levels of TB, DB, and TBA in groups A and B, treated with ^131^I, exhibited significant improvements. A notable finding is the lack of a significant reduction in TBA levels observed in group C. This phenomenon can be attributed to several factors. Unlike groups A and B, where ^131^I treatment effectively lowered thyroid hormone levels. The recovery time of TBA is generally longer than other liver function indexes. Group C received ALSS treatment alone without radioactive iodine therapy. The crux of hyperthyroidism-induced hepatotoxicity lies in the heightened metabolic state triggered by excessive thyroid hormones, fostering liver damage. The accelerated catabolism of liver glycogen and proteins due to thyroid hormones can result in hepatocyte degeneration and immune-related liver injury (23). The underlying cause of hepatotoxicity in hyperthyroidism is attributed to the elevated metabolic state resulting from excessive thyroid hormones, which leads to a cascade of events including free radical damage, hepatocyte degeneration, and autoimmune-related liver damage. While hyperthyroidism commonly causes mild cholestatic liver injury, severe cholestasis syndrome is rare, and controlling hyperthyroidism generally yields improvements in cholestasis symptoms (24). Different severities of hyperthyroidism may impact these mechanisms in various ways. More severe hyperthyroidism could exacerbate metabolic stress, leading to greater oxidative damage and immune-mediated liver injury, while milder forms of the condition might have a relatively lesser impact on liver function. Patients with more severe hyperthyroidism should receive ¹³¹I treatment as early as possible. The intricate interaction between elevated thyroid hormone levels and liver function test results is partly attributed to the liver's crucial role in thyroid hormone metabolism (25). Effective management of liver failure in GD patients largely depends on controlling GD, with ^131^I demonstrating its efficacy and safety.

Group B underwent a combined treatment of ^131^I and ALSS, yielding the highest effectiveness rate at the 2-month mark post-treatment, thus demonstrating favorable outcomes. The mortality rate in group C was elevated compared to the other two groups. This could potentially be attributed to the absence of ^131^I treatment to address hyperthyroidism (26, 27). While ALSS treatment effectively eliminates toxins in liver failure patients, such as serum creatinine, blood urea nitrogen, blood ammonia, and bilirubin, it does not directly address the underlying cause of hepatotoxicity in hyperthyroidism.

ALSS plays a crucial role in treating hyperthyroidism complicated by liver failure by improving liver function and supporting hepatocyte regeneration. Although it does not directly reduce thyroid hormone levels, ALSS effectively removes toxins and metabolic waste, reducing liver damage caused by excessive thyroid hormones. By employing techniques like artificial blood purification and plasma exchange, ALSS decreases metabolic stress on the liver, giving it a better chance to repair. Additionally, it helps maintain essential nutrients and proteins, preventing malnutrition and immune deficiencies, especially in critical situations like a thyroid storm (21). Overall, while ALSS does not target thyroid hormone levels directly, it is essential in managing liver-related complications, making it a key component in the comprehensive treatment of these patients.

^131^I treatment can significantly reduce thyroid hormone levels and establish conducive conditions for the restoration of liver function. Patients with more severe hyperthyroidism should receive ¹³¹I treatment as early as possible. Complications associated with ALSS encompass bleeding, coagulation disorders, hypotension, secondary infections, allergic reactions, metabolic imbalance syndrome, hypercitratemia, and the potential risk of mortality (28). Remarkably, the overall length of hospital stay and total cost for group C were lower than those for group B, primarily due to the mortality of some patients. However, it is noteworthy that the average hospitalization cost for group C was significantly higher, primarily owing to the high expense of ALSS (29). ALSS adjuvant therapy didn't demonstrate evident advantages in this study.

This study has several limitations that should be considered when interpreting the results. First, as a single-center retrospective study, the findings are subject to certain inherent biases and limitations, such as potential selection bias and lack of randomization, which may affect the generalizability of the results to a broader population. Secondly, the study faced challenges in standardizing clinical baseline values across all participants, leading to some variability that could influence the outcomes. Thirdly, the relatively small sample size limits the statistical power of the study, making it difficult to draw definitive conclusions. Therefore, larger, multicenter prospective studies are needed to confirm these findings and provide more robust evidence.

Previous research has established the effectiveness of ALSS in improving liver function and the efficacy of ¹³¹I in treating hyperthyroidism. Additionally, some studies have confirmed the safety and benefits of combining ALSS with ¹³¹I in managing hyperthyroidism complicated by liver failure. However, the interplay between hyperthyroidism and liver failure presents a complex clinical challenge. This article is the first to systematically compare the outcomes of three different treatment approaches, offering valuable insights that could improve treatment cost-effectiveness and clinical outcomes, ultimately benefiting patients facing this difficult condition.

## Conclusion

This study underscores the significance of prioritizing the treatment of hyperthyroidism symptoms in patients grappling with the complexities of concurrent liver failure and hyperthyroidism. The administration of ^131^I treatment for GD resulted in a gradual reduction in thyroid hormone levels. In the context of enhancing liver function, specifically in terms of TB, DB, and TBA, the prompt utilization of ^131^I is recommended to mitigate the adverse impact of hyperthyroidism on liver function and foster a favorable environment for liver function recovery. Although ALSS did not exhibit substantial advantages, we maintain a strong belief in the viability and efficacy of the combined approach involving ^131^I and ALSS. This combined strategy holds promise as a feasible and effective treatment avenue, offering improved outcomes and enhanced disease management for individuals with both hyperthyroidism and liver failure.

## Supplementary Materials

Supplementary Table 1 The effective rate of each time point in the three groups of patients.

Supplementary Table 2 Mortality among the three groups.

Supplementary Table 3 The cost, hospitalization days and daily average cost of the three groups.

## Declaration of interest

The authors declare that there is no conflict of interest that could be perceived as prejudicing the impartiality of the research reported.

## Funding

This work was supported by the General Program of the Chongqing Natural Science Foundation (CSTB2022NSCQ-MSX0901) and the Kuanren Talents Program of the Second Affiliated Hospital of Chongqing Medical Universityhttp://dx.doi.org/10.13039/501100004374 (Kryc-yq-2204).

## Ethical statement

The study has been approved by the Ethics Committee of the Second Affiliated Hospital of Chongqing Medical University (Approval No: 2023.No112). The study was performed per the ethical standards as laid down in the 1964 Declaration of Helsinki.

## Data availability

Data will be made available upon request.

## Author contribution statement

DF: drafting of the paper. SL and CZ: analysis and interpretation of the data. YW and GY: conception and design. HZ and MR: revising it critically for intellectual content. We confirm the final approval of the version to be published. All authors agree to be accountable for all aspects of the work.
