# Supplementary material for: Comparing the prognostic impact of 131I and/or artificial liver support system on liver function failure combined with hyperthyroidism
Source: Endocr Connect. 2024 Oct 7;13(11):e240330. doi: 10.1530/EC-24-0330 (PMC11466263; doi:10.1530/EC-24-0330)
Supplement: Supplementary Table 1 The effective rate of each time point in the three groups of patients. [file supplementary_table_1.pdf]

Supplementary Table 1：The effective rate of each time point in the three groups of patients.

Time	Group	Cases (n)	Cured(n)	Improved (n)	No response (n)	Effective rate	p
One week	GroupA	34	0	22	12	64.71%	0.909
	GroupB	17	0	12	5	70.59%	
	GroupC	23	0	15	8	65.22%	
Discharge	GroupA	34	0	26	8	76.47%	0.821
	GroupB	17	0	12	5	70.59%	
	GroupC	23	0	16	7	69.57%	
Follow-up in two months	GroupA	34	5	22	7	79.41%	0.282
	GroupB	17	3	11	3	82.35%	
	GroupC	23	2	12	9	60.87%	
