# Supplementary material for: Comparing the prognostic impact of 131I and/or artificial liver support system on liver function failure combined with hyperthyroidism
Source: Endocr Connect. 2024 Oct 7;13(11):e240330. doi: 10.1530/EC-24-0330 (PMC11466263; doi:10.1530/EC-24-0330)
Supplement: Supplementary Table 2 Mortality among the three groups. [file supplementary_table_2.pdf]

Supplementary Table 2： Mortality among the three groups.

**Group**	**Death(n)**	**Total (n)**	**Mortality**
GroupA	2	34	5.88%
GroupB	3	17	23.53%
GroupC	9	25	36.00%
