# Supplementary material for: Comparing the prognostic impact of 131I and/or artificial liver support system on liver function failure combined with hyperthyroidism
Source: Endocr Connect. 2024 Oct 7;13(11):e240330. doi: 10.1530/EC-24-0330 (PMC11466263; doi:10.1530/EC-24-0330)
Supplement: Supplementary Table 3 The cost, hospitalization days and daily average cost of the three groups. [file supplementary_table_3.pdf]

Supplementary Table 3：The cost, hospitalization days and daily average cost of the three groups.

	GroupA	GroupB	GroupC	p
Number of ALSS treatments(n)	-	1.94±0.234	2.24±0.358	0.978
Cost (￥)	38203.43±22581.95	111281.15±80338.7	82135.7±53123.19	<0.001
Days of hospitalization(days)	26.97±15.37	40.15±14.52	28.22±16.15	0.026
Daily Cost (￥)	1521.34±799.05	2665.02±1154.29	3308.03±2063.48	<0.001
